# Analysis of Rinsing Fluid during Negative Pressure Wound Therapy with Instillation: A Potential Monitoring Tool in Acute and Chronic Wound Treatment. A Pilot Study

**DOI:** 10.3390/cells10040732

**Published:** 2021-03-26

**Authors:** Christian D. Taeger, Stefan Wallner, Teresa Martini, Daniel Schiltz, Andreas Kehrer, Lukas Prantl, Niklas Biermann

**Affiliations:** 1 Center of Plastic, Hand and Reconstructive Surgery, University Hospital Regensburg, Franz-Josef-Strauß-Allee 11, 93053 Regensburg, Germany; teresa.martini@stud.uni-regensburg.de (T.M.); schiltz.da@gmail.com (D.S.); andreaskehrer@gmx.de (A.K.); lukas.prantl@ukr.de (L.P.); niklas.biermann@ukr.de (N.B.); 2 Institute for Clinical Chemistry, University Hospital Regensburg, Franz-Josef-Strauß-Allee 11, 93053 Regensburg, Germany; stefan.wallner@ukr.de

**Keywords:** negative pressure wound therapy with instillation, wound healing, cytokines

## Abstract

Background: During negative pressure wound therapy (NPWT), open wounds are draped with a nontransparent sponge, making daily wound evaluation impossible. Sometimes, late or undetected bacterial infections and postoperative bleeding result in repetitive surgery, thus prolonging inpatient time. With the introduction of additional fluid instillation (NPWTi), the wound surface is rinsed, and bacteria, proteins and biomarkers are flushed into a collecting canister, which is later discarded. Methods: The aim of this pilot study was to analyze rinsing fluid samples (0.9% sodium chloride) from the NPWTi device in patients with acute and chronic wounds. In 31 consecutive patients a standardized laboratory analysis was performed to evaluate cellular composition and potassium, phosphate, lactate dehydrooxygenase, pH and total protein levels. Results: While there was an increase in the total cellular amount and the number of polymorphonuclear cells, the number of red blood cells (RBC) decreased after surgery. Potassium and pH showed no significant changes in the first three postoperative days, whereas total protein showed an undulant and partially significant course. Conclusion: We were able to quantify cellular metabolites by analyzing the rinsing fluid of NPWTi. We propose the analysis of this material as a novel and potentially promising tool to monitor wound status without removal of the dressing. The establishment of reference values might help to improve the NPWTi therapy.

## 1. Introduction

Despite a steadily increasing application of negative pressure wound therapy with instillation (NPWTi) in acute and chronic wound care, a thorough understanding of the underlying physiology, as well as of monitoring tools during the dressing phase, is lacking [1,2,3,4,5,6,7,8]. In NPWTi, the additional fluid instillation is thought to facilitate the removal of microorganisms, dilute inflammatory and cytotoxic molecules, as well as more strongly influence angiogenesis due to intermittent suction intervals [9,10,11]. This form of negative pressure wound therapy (NPWT) has been shown to be particularly suitable for the treatment of infected wounds, and also leads to improved granulation of the wound bed [11,12]. Proving and monitoring these principles is challenging, since wound healing involves a highly orchestrated sequence of cellular and biomechanical aspects, and clinical evaluation is possible only during dressing changes [13,14]. For decades, the detection and monitoring of pathologies was done through laboratory analysis of human fluids, e.g., blood or urine. In combination with a biochemical routine, new pathologies can be detected, and organ recovery as well as the course of a disease can be mirrored. Wound monitoring has shown that altered levels of proteins, growth factors and systemically administered antibiotics can be detected within the wound exudate [15,16]. The analysis of wound fluid, however, remains controversial, since no consensus exists on the complexity and invasiveness of fluid collection methods. Both invasive and non-invasive methods of gathering material from wounds treated with NPWT show an altered microenvironment and promising cascades, especially in cytokine production [17,18]. Therefore, we analyzed the rinsing fluid during NPWTi in patients with acute and chronic wounds over several days. The aim of this pilot study was to assess whether it was possible to detect metabolites or cytokines within the rinsing fluid, and whether these might be linked to the clinical course.

## 2. Materials and Methods

This is a prospective observational pilot study investigating the rinsing fluid of NPWTi dressings after surgical debridement in order to assess the eligibility of the rinsing fluid to serve as a potential diagnostic and safety tool. It was conducted with ethics approval (18-1264-101) and according to the 2010 CONSORT (Consolidated Standards of Reporting Trials) guidelines in accordance with the Helsinki Declaration. It was registered in a public German trial registry (DRKS00017669). All patients participating in this study provided written informed consent. Between September 2019 and May 2020, 35 consecutive patients with open wounds were screened at our hospital for eligibility into this trial. Inclusion criteria were acute and chronic wounds that made NPWTi necessary to lower the bacterial bioburden prior to reconstruction. All open wounds suitable for NPWTi application were included regardless of their cause, except cancer. Four patients with chronic decubital ulcers were excluded, since the NPWTi device repeatedly disconnected due to close proximity to the anus. A commonly available vacuum system with instillation (V.A.C. VERAFLOTM, KCI Medizinprodukte GmbH, Wiesbaden, Germany) was used in every case, and the instillation volume was manually set in relation to the wound size and kept constant during the entire course of treatment. The rinsing interval was set to three hours and the instillation fluid remained for twenty minutes in contact with the wound bed before removal. The study protocol included the evaluation and analysis of one fluid sample every day always at the same day-time, starting on the day of the first surgical debridement until the wound was closed. Instillation fluid was a standard 0.9% sodium chloride solution (Braun, Melsungen, Germany). Samples were taken out of a special sampling canister provided by KCI without hygroscopic gel and using a sterile syringe. These canisters were only used for a single rinsing interval and removed after the above-mentioned residence time. Immediate laboratory analysis was done for all parameters except the total protein, which was determined later from fresh frozen fluid samples. These were simultaneously stored at −80 °C in commercially available Eppendorf cups (Eppendorf AG, Hamburg, Germany). As a pilot study, the laboratory analysis included the measurements of electrolytes and cellular components. Electrolytes included potassium via an ion-sensitive electrode, phosphate and lactate dehydroxygenase (LDH) via photometry (Dimension Vista 1500, Siemens Healthineers, Erlangen, Germany) and pH via potentiometry (ABL90 flex, Radiometer, Krefeld, Germany). To determine the number of different cells in the fluid sample, flow cytometry (XE5000, Sysmex, Kobe, Japan) and a manual microscopic analysis were performed. The BCA Assay Protein Quantitation Kit (Interchim, Montulocon, France) including optical density measurement (Sunrise, Tecan Trading AG, Männedorf, Switzerland) was used at a wavelength of 562 nm. All measurements were taken according to the manufacturer’s instructions.

Data is presented as the arithmetic median or mean with standard deviation (SD). The Kolmogorov–Smirnov test was used to test variables for normality. Variables that did not exhibit normal distribution were compared using the Wilcoxon test. Variables with normal distribution were not present in our investigation. The Friedman test was not used due to dissimilar sample numbers. A *p*-value < 0.05 was considered significant. The software used to perform the statistical analysis was the “Statistical Package for the Social Sciences” (SPSS Inc., Chicago, IL, USA) Version 26.0.

## 3. Results

### 3.1. Patients

Of the 31 participants in the study, 20 (64.5%) were male and 11 (35.5%) were female. The overall mean age was 63 (SD 16.9). Every participant received a mean of 2 (SD 0.8) debridements prior to reconstruction. The NPWTi device was applied for a mean of 5.4 (SD 2) days on the wound.

Wound volume was estimated during each debridement with a metric ruler and categorized as small (<10 cm^3^) *n* = 5, middle (10–100 cm^3^) *n* = 21 and large (>100 cm^3^) *n* = 5 wounds. The mean wound volume was 59 cm^3^ (SD 84).

The cause of the wound was chronic in 23 patients (ischemic *n* = 2, post-traumatic/post-infection *n* = 12, decubital ulceration *n* = 6 and post-cancer *n* = 3) and acute in eight patients (traumatic *n* = 4, burns *n* = 1 and ischemic/compartment *n* = 3) (Table 1).

### 3.2. Laboratory Analysis

**Figure 1:**
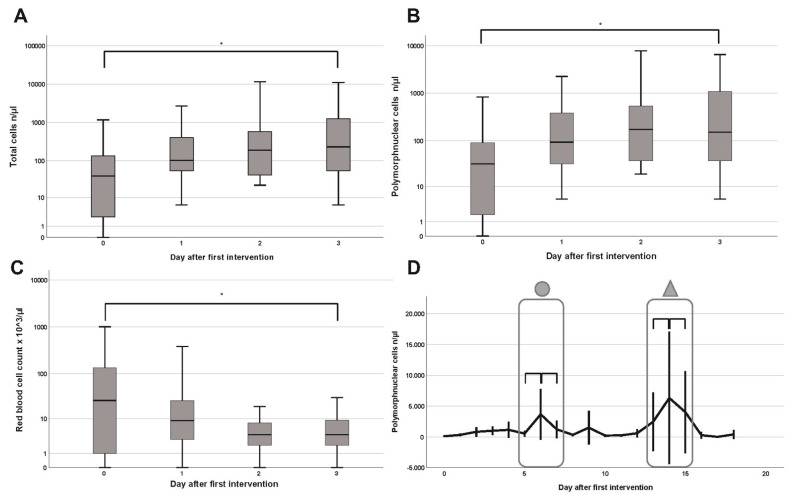
Boxplots of the total cellular amount (n/µL) (**A**), polymorphonuclear cells (PMNs) (n/µL) (**B**) and red blood cell count (n × 10^3^) (**C**) on the day of the operation (0) and first to third postoperative day. (**D**) Entire postoperative course of the PMNs (n/µL) after the first operation. Encircled areas with a ring or triangle on the top mark intervals of second and third operation, respectively. Vertical bars indicate the 95% confidence interval. The asterisk (*) indicates significance.

The standardized laboratory analysis included a cell determination via flow cytometry and manual microscopy, as well as the measurement of potassium, phosphate, LDH, pH levels and the total protein amount. LDH and phosphate remained constant and thus are not shown in our results. After the initial operation, the first four samples were chosen for comparison, as they were available in most of the patients. The total cellular amount as well as the number of polymorphonuclear cells (PMN) increased from the first to the fourth measurement from a median of 39/µL and 32/µL to 229/µL and 150/µL, respectively (*p* = 0.02 and *p* = 0.03) (Figure 1A,B). A long-term course of the PMNs showed a gradual increase after the initial operation, peaking around the sixth day (*p* = 0.42). After a drop around day eight, another steady increase was noted, again peaking around day 14 (*p* = 0.66). Both peaks occurred within the interval of the second and third operation (Figure 1D). The red blood cell count showed a decrease from a median of 26 to 4/µL when measured on day three (*p* = 0.02) (Figure 1C).

**Figure 2:**
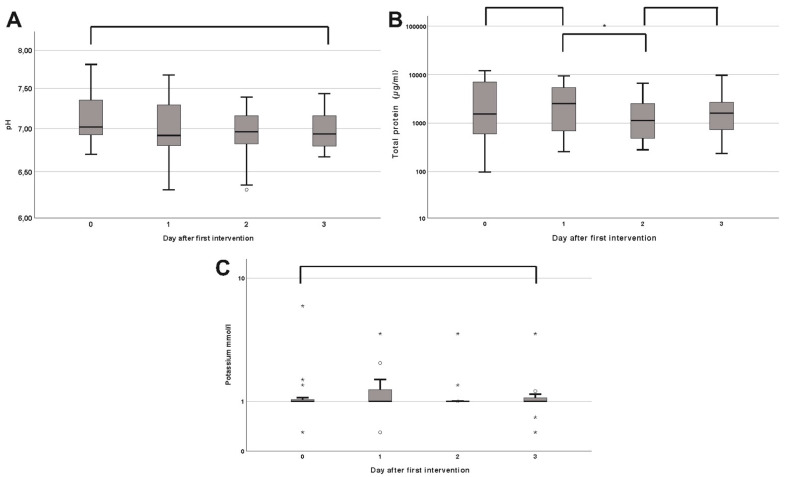
Boxplots of the pH-value (**A**), potassium (mmol/L) (**C**) and total protein (µg/mL) (**B**) on the day of the operation (0.) and first to third postoperative day. Stars and circles indicate extreme values exceeding the 95% confidence interval. The asterisk (*) indicates significance.

The pH value showed a non-significant decrease from a median of 7.02 in the first measurement to 6.99 three days postoperatively (*p* = 0.67) (Figure 2A). The median potassium level remained nearly constant at around 0.99 mmol/L (*p* = 0.46) (Figure 2C). Protein levels showed an undulant course with an initial increase in the median to 2.549 µg/mL (*p* = 0.19) on day one, followed by a decrease in the median to 1.139 µg/mL (*p* = 0.02) and 1.596 µg/mL (*p* = 0.15) on days two and three, respectively (Figure 2B).

## 4. Discussion

Vacuum therapy is undoubtedly an effective tool in the treatment of acute and chronic wound conditioning [19,20]. However, the underlying mechanisms are not conclusively understood and clinical monitoring between dressing changes is limited.

In an attempt to further contribute to the understanding of NPWT(i) and establish a monitoring tool for the clinical routine during the NPWT treatment phase, we analyzed the rinsing fluid of NPWTi devices in a pilot study. We were able to quantify significant levels of potassium, phosphate, lactate dehydroxygenase (LDH), pH and the amount of protein as well as identify different cells in the disposed rinsing fluid. As locally produced biomarkers, these parameters might provide additional information on the wound status without changing the dressing.

Previous studies have suggested that wound fluid is a systemic mirror containing important information about the inflammatory response. Polykandriotis et al. analyzed the wound exudate in NPWT draining lines and measured the levels of systemically administered antibiotics. They were able to detect almost two thirds of the plasma concentration in the draining fluid one hour after intravenous administration [15]. Moues et al. investigated the influence of NPWT on the extracellular collagenous matrix components and used a polyvinylidene fluoride filter to collect the wound fluid. Significant levels of albumin, matrixmetalloproteinases and tissue inhibitors were found as an expression of biochemical markers reflecting parts of the intracellular response [21]. It is not yet clear how precisely the diluted wound exudate mirrors the healing cascade, and, in contrast to invasive punch biopsies, a complete genomic and proteomic evaluation remains challenging.

However, we were able to quantify significant levels of various electrolytes and cell components in the rinsing fluid of NPWTi devices. Several interesting observations are possible, since there was a significant decline in the RBC count on the days following the first operation. This aligns the initial surgical debridement with careful blood coagulation afterwards. The appearance of increased PMNs and the total cellular amount might resemble the beginning of the inflammatory tissue response. Granulocytes are usually not observed within the normal skin, but since their primary goal is to defend the skin from infections, they are the first circulating cells to move to the incisional wound, probably following chemotaxis and extensive local cytokine production [22]. The measured pH value was rather high and comparable to acute split-thickness skin graft donor sites [23] or chronic wounds after radiotherapy [24]. Especially in cutaneous wounds, many parts of the healing cascade (e.g., enzymes like matrixmetalloproteinase and collagenase) are pH-dependent and the pH gradient in the wound bed alters the cellular function [25,26]. A skin surface pH (ss-pH) ranges between 5.4 and 5.9 and gradually increases with depth [27]. With respect to the wounds investigated in our study, the skin integrity was destroyed in the vast majority of cases, and regardless of the wound genesis, the surgical debridement induced an acute wound status. This might explain the rather high pH value in the physiological pH of the rinsing fluid itself. Additionally, a unification of the wound pH by the rinsing fluid should be discussed as a possible mechanism of action. Continuous pH monitoring might serve as a tool for assessing the status of skin granulation. Regarding the electrolytes, the median potassium level showed a non-significant increase on the first day after the initial debridement. As a predominantly intracellular electrolyte, the surgical removal of infected and inflamed tissue inevitably destroys cells and releases potassium, again serving as a potential monitoring asset. Another metabolite that possibly discloses information on the cellular barrier function is the total amount of protein. The interpretation is challenging, since individual amounts of up to 1 g/mL were found. When compared to renal function, protein levels as high as 1 g/mL over 24 h would depict proteinuria in severe nephrotic syndrome, indicating damage to the glomerular barrier. With a missing epidermal barrier, however, such high protein levels might indicate the beginning of the inflammatory healing phase. Finally, to show the sensitivity of the fluid evaluation, we provided a 17-day, long-term profile of the PMNs. By analyzing the spikes alone, one can assume a preceding debridement, since 95% of the second and third surgeries were conducted just before the two peaks. This study has several strengths and limitations. As a pilot study, only preliminary data is presented, and reference values are lacking. Therefore, the comparability to other studies is limited, plus the decreasing and heterogeneous number of fluid samples limits the statistical power in the long-term analysis. Additionally, the overall cohort size is rather small and lacks a control group. However, as a hypothesis-generating pilot study with different endpoints, the chosen case number seems sufficient to reliably estimate the statistics with missing reference values. Since the exact composition of the rinsing liquid (0.9% sodium chloride) is known, the absence of a distinct control group should be of minor importance. Another strength is the immediate processing of the fluid samples and the use of already established laboratory tools limiting methodological bias. As a next step, further evaluation of cytokines and extracellular metabolites might allow additional insight into the healing cascade and validate fluid analysis as a beneficial monitoring tool. The monitoring aspects may also be implemented into the clinical routine, e.g., continuous RBC measurement to detect rebleeding to increase patient safety. Additionally, the wound status may be monitored in terms of superinfections by increasing PMNs.

## 5. Conclusions

We were able to detect and quantify cells as well as biochemical markers in the rinsing fluid of NPWTi devices. We propose the analysis of this material as a novel and very promising tool to monitor wounds and to investigate the inflammatory tissue response without removal of the dressing. Future investigations should address larger patient cohorts and other cytokines to potentially establish individualized NPWT(i) settings.

## Figures and Tables

**Table 1:** Patient and wound characteristics.

Gender	Wound Size	Chronic Cause	Acute Cause
Male *n* = 20 Female *n* = 11	Small (<10 cm^3^) *n* = 5	*n* = 23	*n* = 8 burns *n* = 1
	Medium (10–100 cm^3^) *n* = 21 Large (>100 cm^3^) *n* = 5	ischemic *n* = 2 post-traumatic/ post-infection *n* = 12 decubital ulceration *n* = 6 carcinoma *n* = 3	ischemic/compartment *n* = 3 traumatic *n* = 4

## Data Availability

Data is contained within the article.
